# Accuracy of endometrial sampling in the diagnosis of endometrial cancer: a multicenter retrospective analysis of the JAGO-NOGGO

**DOI:** 10.1186/s12885-024-12127-7

**Published:** 2024-03-25

**Authors:** Zaher Alwafai, Maximilian Heinz Beck, Sepideh Fazeli, Kathleen Gürtler, Christine Kunz, Juliane Singhartinger, Dominika Trojnarska, Dario Zocholl, David Johannes Krankenberg, Jens-Uwe Blohmer, Jalid Sehouli, Klaus Pietzner

**Affiliations:** 1 Department of Gynecology and Obstetrics, University of Greifswald, Greifswald, Germany; 2 Young Academy of Gynecologic Oncology (JAGO), Berlin, Germany; 3 Department of Gynecology With Center for Oncological Surgery, Charité-Universitätsmedizin Berlin, corporate member of Freie Universität Berlin, Humboldt-Universität Zu Berlin, Berlin Institute of Health, Charité Universitätsmedizin Berlin, Campus Virchow Klinikum, Berlin, Germany; 4 Department of Gynecology With Breast Center, Charité-Universitätsmedizin Berlin, corporate member of Freie Universität Berlin, Humboldt-Universität Zu Berlin, Berlin Institute of Health, Charité Universitätsmedizin Berlin, Campus Mitte, Berlin, Germany; 5 Klinik Für Gynäkologie, Krankenhaus Waldfriede, Berlin, Germany; 6 Klinik Für Gynäkologie, DRK-Kliniken Berlin Westend, Berlin, Germany; 7 Department of Gynecology and Obstetrics, Krankenhaus St. Elisabeth Und Barbara, Halle, Germany; 8 Department of Gynecology and Obstetrics, Klinikum Traunstein, Traunstein, Germany; 9 Faculty of Health Sciences, Jagiellonian University Medical College, Cracow, Poland; 10 Institute of Biometry and Clinical Epidemiology, Charité - Universitätsmedizin Berlin, corporate member of, Freie Universität Berlin and Humboldt-Universität Zu Berlin, Berlin, Germany

**Keywords:** Endometrial cancer, Endometrial sampling, Accuracy, Diagnosis

## Abstract

**Background:**

Accurate preoperative molecular and histological risk stratification is essential for effective treatment planning in endometrial cancer. However, inconsistencies between pre- and postoperative tumor histology have been reported in previous studies. To address this issue and identify risk factors related to inaccurate histologic diagnosis after preoperative endometrial evaluation, we conducted this retrospective analysis.

**Methods:**

We conducted a retrospective analysis involving 375 patients treated for primary endometrial cancer in five different gynaecological departments in Germany. Histological assessments of curettage and hysterectomy specimens were collected and evaluated.

**Results:**

Preoperative histologic subtype was confirmed in 89.5% of cases and preoperative tumor grading in 75.2% of cases. Higher rates of histologic subtype variations (36.84%) were observed for non-endometrioid carcinomas. Non-endometrioid (OR 4.41) histology and high-grade (OR 8.37) carcinomas were identified as predictors of diverging histologic subtypes, while intermediate (OR 5.04) and high grading (OR 3.94) predicted diverging tumor grading.

**Conclusion:**

When planning therapy for endometrial cancer, the limited accuracy of endometrial sampling, especially in case of non-endometrioid histology or high tumor grading, should be carefully considered.

## Introduction

Endometrial cancer is ranked as the sixth most prevalent cancer in women worldwide and exhibits the highest incidence rates among gynecological malignancies in developed countries [1, 2]. In recent decades, a significant increase in the occurrence of endometrial cancer was observed, which could be primarily attributed to the rising prevalence of metabolic syndrome and obesity in the affected regions [1–4]. Predictions indicate a further rise in the incidence of endometrial cancer within the upcoming years [5]. Given these trends, early detection and accurate preoperative diagnostic of endometrial cancer and customization of therapeutic strategies are needed. Currently, the primary diagnostic strategy for evaluating suspected endometrial cancer involves various endometrial sampling techniques, including curettage, hysteroscopic guided curettage, or endometrial biopsy [6]. These procedures not only aid in the detection of invasive disease but also provide valuable information on molecular aberrations, histomorphologic subtypes and tumor grading, which can facilitate further risk stratification [7–9]. The conventional histopathological classification proposed by Bokhman categorizes endometrial cancer into estrogen-dependent low-grade type I and non-estrogen-dependent high-grade type II carcinomas [10]. This system has faced increasing challenges with the improved understanding of underlying molecular aberrations, such as p53 mutations, mismatch repair deficiency or POLE mutations [7, 11, 12]. Nonetheless the identification of histomorphological subtypes and grading, is still present in clinical routine and is widely used for preoperative risk stratification [6, 11, 13, 14]. Numerous studies reported inconsistencies between preoperative and postoperative tumor histology [15], which could lead to insufficient treatment and unfavorable outcomes as failure of detecting high-risk carcinomas was associated with adverse outcomes in retrospective cohort studies [16]. To comprehensively assess the precision of endometrial sampling within the German healthcare system and identify potential risk factors associated with inadequate histologic diagnosis following preoperative endometrial evaluation, we conducted this multicenter retrospective analysis.

## Methods

### Objectives and end points

We performed a retrospective multicenter analysis aimed at assessing the precision of endometrial sampling in individuals diagnosed with endometrial cancer. Our secondary objective was to identify potential risk factors associated with discrepancies in histology between curettage and hysterectomy specimens. The present study was approved by the local ethics committee of the university hospital of Greifswald (BB 109/22). In accordance with the decision of the ethics committee, no informed consent was obtained due to the retrospective design of the analysis.

### Patients and data collection

Data of patients with surgical treated primary endometrial cancer were included in five participating German gynecological departments in this multicentric retrospective analysis between January 2013 and December 2015. All patients underwent a prior endometrial evaluation in form of curettage, hysteroscopy guided curettage or endometrial biopsy followed by total hysterectomy, whether vaginal, laparoscopic or per laparotomy. Patients with simultaneous gynecological malignancies were not included in this study.

Patient’s data and clinicopathological information were retrospectively obtained from the respective hospital data system. We collected information regarding the pre- and postoperative histopathological subtype and grading, TNM classification, age, menopausal status, type of surgery, interval between curettage and hysterectomy and the respective institution of pathology. Incomplete data sets were excluded.

The project was developed as part of a scientific and clinical fellowship program of the JAGO – the Young Academy of Gynecologic Oncology (“Junge Gynäkologische Onkologie”—JAGO) of the Northeastern German Society of Gynecologic Oncology (“Die Nord-Ostdeutsche Gesellschaft für Gynäkologische Onkologie” – NOGGO e.V.) under advice and with the input of interprofessional and interdisciplinary experts.

### Data analysis

Before evaluation, data was checked for plausibility. Descriptive statistics were applied to report results of histopathological evaluation of endometrial sampling and hysterectomy, focusing on histological subtypes and tumor grading. The histological subtypes considered for analysis included endometrial, serous, carcinosarcoma, clear cell, and other subtypes. Due to limited numbers, additional histotypes were grouped under the category of 'others'.

The accuracy outcomes assessed included sensitivity, specificity, positive predictive value (PPV) and negative predictive value (NPV), for both preoperatively assessed tumor grading and histological subtypes. Cases of endometrial hyperplasia after endometrial biopsy with subsequent evidence of endometrial carcinoma in the hysterectomy specimen were not included in the accuracy outcomes analysis.

Group differences were evaluated using two-sided t-testing or ANOVA. A multiple regression analysis using a logistic regression model was applied to identify potential risk factors for diverging histopathological results between preoperative and final histopathological results, each for grading and histologic subtype. Data is presented as mean ± standard deviation (SD) or absolute and relative frequencies depending on scale, if not stated otherwise. A *p* value < 0.05 was considered to indicate statistical significance. All *p* values constitute exploratory data analysis. Statistical analysis was performed using SPSS Version 28 ® (IBM Corp./ USA).

## Results

### General characteristics

A total of 375 patients from five different gynecologic departments were included in the present retrospective analysis. The majority of patients was treated for tumors in early stage of disease. The participants had an average age of 66.53 ± 12.01 years. Among the five participating departments, two were affiliated with universities, while the remaining three were non-university institutions. The average time elapsed between endometrial sampling and hysterectomy was found to be 33.9 ± 40.8 days. In 28.3% of the cases, the specimens obtained from endometrial biopsy and hysterectomy underwent evaluation at different pathology institutions. For a more detailed overview of clinical characteristics and surgical procedures, please refer to Table 1.

**Table 1:** Clinical characteristics. Data is presented as percentage (number). LAVH = Laparoscopic assisted Vaginal Hysterectomy, d = days

***n***	375
**Age (years)**	66.53 ± 12.01
**Menopausal status**	
* Premenopausal*	7.2% (27)
* Perimenopausal*	3.2% (12)
* Postmenopausal*	89.6% (336)
**Type of Hysterectomy**	
* Abdominal Hysterectomy*	36.5% (137)
* Vaginal Hysterectomy*	12.0% (45)
* Laparoscopic Hysterectomy*	11.5% (43)
* LAVH*	39.5% (148)
* Not specified*	0.6% (2)
**Same Institute of Pathology**	
*Yes*	69.9% (262)
*No*	28.3% (106)
**Interval Biopsy-Hysterectomy (d)**	33.9 ± 40.8
**TNM—Tumor (T)**	
* T1a*	49.9% (187)
* T1b*	24.8% (93)
* T2*	10.4% (39)
* T3a*	3.7% (14)
* T3b*	5.9% (22)
* T4a*	0.8% (3)
**TNM—Nodal status (N)**	
* N0*	35.2% (132)
* N1*	9.1% (34)
* N2*	0.8% (3)
* Nx*	54.9% (206)
**TNM—Metastatic disease (M)**	
* M0*	58.4% (219)
* M1*	1.6% (6)
* Mx*	40.0% (150)

### Histological characterization

 The majority of tumors exhibited endometrial histology and were characterized by a low tumor grading. Among the cases diagnosed with endometrial carcinoma following endometrial sampling, 84.0% displayed endometrioid histology. Serous histology accounted for the second most frequent histologic subtype, representing 5.9% of cases, followed by carcinosarcoma (3.7%) and clear cell carcinoma (2.1%). Less common tumor entities were grouped under the category 'other' to provide a comprehensive overview. This category included mucinous, squamous, transitional, undifferentiated, and mixed-cell tumors. We acknowledge that many mucinous endometrial carcinomas are considered to be endometrioid tumors with mucinous differentiation. Nevertheless, due to the initial diagnosis of mucinous subtype, which included mucinous tumors of gastrointestinal type, these tumors were grouped under the category "Other". For a more detailed overview of the distribution of histologic subtypes, please refer to Table 2.

**Table 2:** Grading and histologic subtypes obtained from endometrial biopsy and hysterectomy. Data is presented as percentage (number)

	Endometrial Biopsy	Hysterectomy
**Grading**		
* G1*	41.3% (155)	43.2% (162)
* G2*	26.4% (99)	32.0% (120)
* G3*	20.0% (75)	24.3% (91)
* Gx*	12.3% (46)	0.5% (2)
**Histologic Subtype**		
* Endometrioid*	76.3% (286)	84.0% (315)
* Serous*	6.9% (26)	5.9% (22)
* Clear cell*	2.1% (8)	2.1% (8)
* Carcinosarcoma*	2.1% (8)	3.7% (14)
* Other*	5.1% (19)	4.3% (16)
* Endometrial hyperplasia*	6.1% (23)	
* Not reported*	1.3% (5)	

In our analysis, we identified 23 cases with detection of atypical endometrial hyperplasia after endometrial sampling and evidence of an invasive endometrial carcinoma in the later hysterectomy specimen. Out of these cases, almost all (*n* = 22/23) exhibited endometrioid histology and one case demonstrated serous histology. In three cases, grading (G1 *n* = 3/3) was also reported when atypical endometrial hyperplasia was detected. Cases with proof of atypical endometrial hyperplasia after endometrial evaluation were not included in the subsequent multivariate analysis. In five cases histologic subtype of endometrial biopsy was not reported. These cases were also not included in the subsequent analysis.

### Accuracy of histologic subtype

Following the confirmation of invasive disease after endometrial sampling, the histologic subtype was successfully established in 89.5% (*n* = 307) of cases. Endometrioid carcinomas demonstrated a comparable high sensitivity of 94.42% and a correspondingly high positive predictive value of 96.10%. However, they exhibited lower specificity (80.35%) and negative predictive value (73.77%). In contrast, non-endometrioid carcinomas displayed lower sensitivity (60%) and positive predictive values (59.02%), but higher specificity (91.26%) and negative predictive values (92.55%). Notably, among the non-endometrioid histologic subtypes, clear cell carcinomas exhibited the highest specificity and negative predictive value, while carcinosarcomas had the lowest sensitivity. For detailed accuracy calculations for the respective histologic subtypes, please refer to Table 3.

**Table 3:** Accuracy analysis for each histologic subtype and grading after endometrial sampling in comparison to the histology of the hysterectomy specimen. A summarized analysis is displayed for non-endometrioid subtypes. PPV = positive predictive value, NPV = negative predictive value

	Specificity	Sensitivity	PPV	NPV
**Grading**				
* G1*	84.73%	83.91%	79.47%	88.21%
* G2*	90.87%	65.52%	78.35%	83.94%
* G3*	96.91%	74.71%	89.04%	91.94%
**Histologic Subtype**				
* Endometrioid*	80.35%	94.42%	96.10%	73.77%
* Non-Endometrioid*	91.26%	60.00%	59.02%	92.55%
* Serous*	96.59%	75.00%	57.69%	98.42%
* Clear cell*	98.23%	75.00%	75.00%	99.40%
* Carcinosarcoma*	98.20%	46.15%	75.00%	97.91%
* Other*	97.25%	60.00%	47.37%	98.15%

**Fig. 1:**
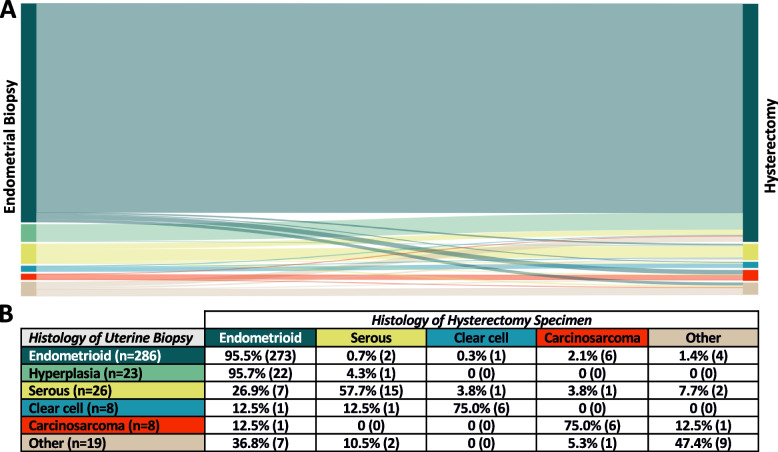
Comparison of histologic subtypes between endometrial sampling and hysterectomy specimens. **A** The Sankey flow diagram illustrates the distribution of histologic subtypes after endometrial sampling on the left side and the corresponding final histologic subtypes observed in the hysterectomy specimens on the right side. The lines represent the transition of histologic subtypes, with the thickness of each line indicating the number of patients. The histologic subtypes are color-coded as follows: Dark green: Endometrioid histology, Bright green: Endometrial hyperplasia, Yellow: Serous histology, Blue: Clear cell histology, Red: Carcinosarcoma, Brown: Other. **B** The table displays the percentage and number of cases for each histologic subtype observed after endometrial sampling (rows) and the corresponding final histologic subtype identified in the hysterectomy specimens (columns)

Consistent with the accuracy analysis, there was a significantly higher frequency of histologic subtype changes observed for non-endometrioid histologic subtypes compared to endometrioid carcinomas (endometroid 5.24% vs. non endometrioid 36.84%; two-sided *p* < 0.001). Figure 1 presents the specific transformations from endometrial biopsy to the final histology observed in the hysterectomy specimens.

### Accuracy of tumor grading

**Fig. 2:**
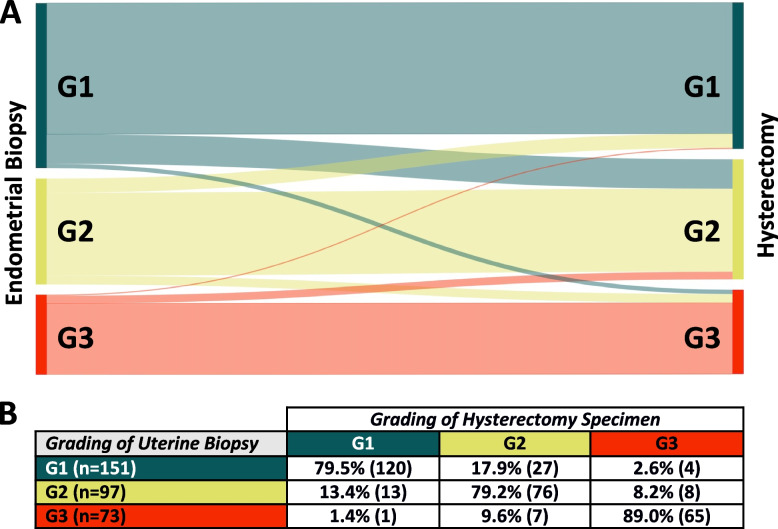
Comparison of tumor grading between endometrial sampling and hysterectomy specimens. **A** The Sankey flow diagram illustrates the distribution of tumor grading after endometrial sampling on the left side and the corresponding final grading observed in the hysterectomy specimens on the right side. The lines represent the transition of grading, with the thickness of each line indicating the number of patients. The respective gradings are color-coded as follows: Dark green: G1, Yellow: G2, Red: G3. **B** The table displays the percentage and number of cases for each tumor grading observed after endometrial sampling (rows) and the corresponding final grading identified in the hysterectomy specimens (columns). Cases of endometrial hyperplasia and not specified histological subtype are not reported in this graph

The overall agreement of tumor grading was 75.2%. Notably, as the tumor grading increased, a higher specificity was observed. Conversely, low-grade tumors (G1) exhibited the highest sensitivity. For a comprehensive overview of the accuracy analysis, please refer to Table 3. Specific transformations from preoperative grading to the final grading observed in the hysterectomy specimens are presented in Fig. 2.

### Risk factors for inaccurate results of endometrial sampling

When specimens of endometrial sampling and hysterectomy were examined in different pathology institutions, a higher occurrence of diverging histologic subtype was observed compared to cases where the specimens were examined in the same institution. However, this difference fell just short of statistical significance (15.79% for different institutions vs. 8.68% for the same institution; univariate two-sided *p* = 0.057). Similarly, no statistically significant difference was observed in tumor grading between different examining pathology institutions (30.10% for different institutions vs. 20.80% for the same institution, *p* = 0.070). In an additional analysis, we investigated whether there existed a disparity in concordance between academic and non-academic institutions. For both tumor grading (two-sided *p* = 0.640) and histological subtype (two-sided *p* = 0.729), no statistically significant differences were detected between academic and non-academic institutions.

To assess the accuracy of histologic subtype and tumor grading, a further analysis was conducted using a logit regression model. Multiple factors were investigated, including patient age, histologic subtype, tumor grading, time elapsed between endometrial biopsy and hysterectomy, and whether the specimens were examined in the same or different pathology institutions. The analysis revealed that cases with non-endometrioid histology (OR 4.41) and high tumor grading (G3) (OR 8.37) had significantly increased odds of having a diverging histologic subtype. Noteworthy, confidence intervals for non-endometroid histologic subtypes and G3 grading were found to be wide, which may indicate problems in the calculation due to small sample size, Table 4. The previously mentioned difference between the examining institutions of pathology did not emerge as a significant predictor in the logit regression analysis. In terms of diverging tumor grading, the identified predictors were intermediate and high tumor grading (G2: OR 5.04; G3: OR 3.94). No other variables examined demonstrated a significant association with diverging histology or tumor grading. For detailed results of the logarithmic regression analysis, please refer to Table 4.

**Table 4:** Results of Logit Regression Analysis for Accuracy of Histologic Subtype and Tumor Grading after endometrial evaluation. Odds ratios (OR) and their corresponding 95% confidence intervals (CI) are provided for each examined predicting variable in the logarithmic regression analysis of histologic subtype and tumor grading accuracy after endometrial sampling

	**Histologic subtype**				**Grading**			
		95%-CI				95%-CI		
	OR	Lower	Upper	*p*	OR	Lower	Upper	*p*
**Sociodemographic**								
* Age*	0.99	0.95	1.03	0.65	0.99	0.96	1.02	0.56
**Pathology**								
* Different Institution*	1.77	0.55	5.73	0.34	1.76	0.76	4.12	0.15
* Interval Biopsy-HE (d)*	1.0	0.99	1.01	0.85	1.01	0.99	1.02	0.13
**Histologic Subtype**								
* Endometriod*	1				1			
* Non-Endometrioid*	**4.41**	1.35	14.41	0.014	0.49	0.14	1.78	0.28
**Grading**								
* G1*	1			0.04	1			0.001
* G2*	2.61	0.46	14.93	0.28	**5.04**	2.09	12.17	< 0.001
* G3*	**8.37**	1.47	47.66	0.02	**3.94**	1.29	12.00	0.016

## Discussion

### Summary of results

In this retrospective multicentric analysis of 375 patients from five different German gynecological departments, the accuracy of endometrial sampling was evaluated. Histologic subtype was confirmed in 89.5% of cases. Non-endometrioid carcinomas had a significantly higher rate of histologic subtype changes compared to endometrioid carcinomas. Non endometrioid histology and higher tumor grading were identified as significant predictor of diverging histologic subtype, while intermediate and high tumor were significant predictors of diverging tumor grading.

### Comparison of the results with the literature

In our analysis, we observed significant variation in agreement between the histological findings of preoperative and hysterectomy, particularly across non endometrioid subtypes. Our findings are consistent with previously published reports, which have shown higher accuracy rates for endometrioid cancers [15, 17]. Some published studies have reported agreement rates of up to 96% for endometrioid histology [17, 18], with the highest agreement rates observed in low-grade endometrioid cancer [16–20]. On the other hand, lower accordance rates have been reported for non-endometrioid carcinomas [18]. Previous studies have indicated that intermediate-grade (G2) tumors tend to exhibit lower sensitivity and accuracy rates, while low-grade tumors demonstrate higher sensitivity rates [16–21]. Consistent with these reports, our multivariate analysis identified intermediate (G2) grading as a significant predictor of divergent tumor grading. It should be noted that G1 and G2 can be grouped together as “low-grade” carcinomas and are treated in the same way. The change between G1 and G2 is not clinically relevant, rather the change from low- to high-grade and vice versa.

Changes of histologic subtype were reported more frequently, when preoperative and final pathology evaluation were carried out in different pathology institutions. However, the specified significance level narrowly missed reaching statistical significance in the two-tailed test (*p* = 0.057). Interestingly, several studies have reported poor interobserver viability in endometrial cancer, particularly for high-grade tumors [22–24]. Notably, these studies focused on interobserver variability within single institutions. It is plausible that interobserver variability might be greater when the endometrial biopsy sample is not concurrently available during the examination of the hysterectomy specimen. This could potentially account for observed interinstitutional variabilities.

### Clinical implications

The surgical approach for treating endometrial cancer is still highly determined by the histopathological risk profile. For non-endometrioid (type II) cancers more radical approaches are recommended due to the worse prognosis [25]. Both national and international guidelines recommend additional omentectomy in patients with serous carcinomas and consider systematic pelvic and para-aortic lymphadenectomy in patients with high-risk endometrial carcinomas [6, 13, 14]. The benefit of lymphadenectomy or sentinel node biopsies in non-endometrial cancer is still a subject of ongoing debate, as retrospective data suggest that adding para-aortic lymphadenectomy may be associated with improved outcomes in type II cancer [26].

Preoperative misdiagnosis in terms of histological subtype and tumor grading might increase the risk of surgical over- or undertreatment, especially in patients with non-endometrioid carcinomas considering the moderate accuracy of histological assessment after endometrial biopsy in this collective. In contrast, molecular profile assessment including p53, POLE, or mismatch-repair deficiency exhibit higher accuracy rates [12, 27–29]. Interobserver agreement in histotyping has been shown to be influenced by the molecular subtype, revealing the lowest concordance rates in p53-mutated endometrioid carcinomas [30]. Significant variations are also reported for tumors with POLE mutations or mismatch repair deficiencies [30]. Specifically, histotyping POLE-mutated endometrial cancers can be challenging due to their frequently heterogeneous histology, which may include high-grade features. Clinically, these tumors have a very favorable prognosis and should be accurately identified as such at the time of diagnosis.

For effective clinical risk stratification, it is crucial to reliably identify high-risk constellations, such as a p53 mutation, as well as low-risk constellations, such as a POLE mutation. Therefore, molecular risk stratification should be performed after endometrial biopsy [31] and before any therapeutic surgical interventions are planned in endometrial cancer. This approach aims to enhance diagnostic accuracy and minimize the risk of surgical over- or undertreatment, particularly in cases of non-endometrioid histology.

### Limitations

The present study has some limitations, which we would like to point out here. Primarily, the retrospective nature of the study should be noted. The data were acquired prior to the incorporation of molecular classification for endometrial cancer into routine clinical practice. Consequently, information regarding mismatch repair status, p53 mutation, polymerase-Ɛ mutation was not systematically documented within our study cohort. Hormone receptor status and proliferation fraction were also not systematically assessed. Given that the primary objective of this study was to underscore the diagnostic uncertainty of endometrial sampling, it would be interesting whether the accuracy in diagnosing histological subtype or tumor grading can be enhanced by considering immunohistochemistry markers, such as p53, ki67 or mismatch repair status. This aspect should be subject to evaluation in subsequent studies.

Additionally, it is noteworthy that we did not assess the expertise and specialization of the examining pathologist, whether they were a trained gynecologic pathologist or a general pathologist. Consequently, our study does not provide insight into whether a gynecologic pathologist with appropriate expertise performs better in diagnosing histologic subtypes or grading in endometrial sampling. However, our study investigated whether there are differences between academic and non-academic institutions. Although a higher volume and a higher degree of specialization can be assumed in academic institutions, our findings revealed no discernible difference in the accuracy of endometrial sampling. It may be speculated that even specialized institutions encounter challenges in achieving precise preoperative diagnosis of histologic subtype and tumor grading.

## Conclusions

This analysis underlines the significance of precise histologic subtype determination and the potential influence of different pathology institutions on subtype consistency. The lower sensitivity of histological assessment in non-endometrioid carcinomas should be considered during treatment planning. Our data indirectly expose the importance and support the additional utilization of preoperative molecular profile assessment of endometrial carcinoma after endometrial evaluation.

## Data Availability

The data presented in this study are available on request from the corresponding author.

## Abbreviations

JAGO: Young Academy of Gynecologic Oncology (“Junge Gynäkologische Onkologie”)
NOGGO: UNortheast German Society of Gynecologic Oncology (“Die Nord-Ostdeutsche Gessellschaft für Gynäkologische Onkologie” NOGGO e.V.)
PPV: Positive Predective Value Northeast German Society of Gynecologic
NPV: Negative Predective Value
