# Correction: Accuracy of endometrial sampling in the diagnosis of endometrial cancer: a multicenter retrospective analysis of the JAGO-NOGGO

**DOI:** 10.1186/s12885-024-12200-1

**Published:** 2024-04-03

**Authors:** Zaher Alwafai, Maximilian Heinz Beck, Sepideh Fazeli, Kathleen Gürtler, Christine Kunz, Juliane Singhartinger, Dominika Trojnarska, Dario Zocholl, David Johannes Krankenberg, Jens-Uwe Blohmer, Jalid Sehouli, Klaus Pietzner

**Affiliations:** 1 Department of Gynecology and Obstetrics, University of Greifswald, Greifswald, Germany; 2 Young Academy of Gynecologic Oncology (JAGO), Berlin, Germany; 3 Department of Gynecology with Center for Oncological Surgery, Charité-Universitätsmedizin Berlin, Corporate Member of Freie Universität Berlin, Humboldt-Universität Zu Berlin, Berlin Institute of Health, Charité Universitätsmedizin Berlin, Campus Virchow Klinikum, Berlin, Germany; 4 Department of Gynecology with Breast Center, Charité- Universitätsmedizin Berlin, Corporate Member of Freie Universität Berlin, Humboldt-Universität Zu Berlin, Berlin Institute of Health, Charité Universitätsmedizin Berlin, Campus Mitte, Berlin, Germany; 5 Klinik Für Gynäkologie, Krankenhaus Waldfriede, Berlin, Germany; 6 Klinik Für Gynäkologie, DRK-Kliniken Berlin Westend, Berlin, Germany; 7 Department of Gynecology and Obstetrics, Krankenhaus St. Elisabeth Und Barbara, Halle, Germany; 8 Department of Gynecology and Obstetrics, Klinikum Traunstein, Traunstein, Germany; 9 Faculty of Health Sciences, Jagiellonian University Medical College, Cracow, Poland; 10 Institute of Biometry and Clinical Epidemiology, Charité-Universitätsmedizin Berlin, Corporate Member of Freie Universität Berlin and Humboldt-Universität Zu Berlin, Berlin, Germany

**Correction: BMC Cancer 24, 380 (2024)**

10.1186/s12885-024-12127-7.

Following publication of the original article [1], the authors noticed a typesetting error, whereby the author, Christine Kunz’s family name was merged with the affiliation. This has been corrected in this article.

The original article [1] has been corrected.
